# Report of treatment intensity and survival outcomes in older patients with glioblastoma diagnosed according to WHO CNS 5 classification

**DOI:** 10.1007/s11060-026-05628-x

**Published:** 2026-06-04

**Authors:** Sophie Therese Williams, Sarah Kingdon, Ola Rominiyi, Cressida Lorimer, Edward Chandy, Mareike Thompson, Ciaran Scott Hill, Giles Critchley, Stephen David Robinson

**Affiliations:** 1 Division of Clinical Medicine, School of Medicine and Population Health, University of Sheffield, Sheffield, UK; 2 Weston Park Cancer Centre, Sheffield Teaching Hospitals Foundation Trust, Sheffield, UK; 3 Tessa Jowell Brain Cancer Mission, London, UK; 4 NHS Lothian, Edinburgh Cancer Centre, Edinburgh, UK; 5 NHS Greater Glasgow and Clyde, Beatson West of Scotland Cancer Centre, Glasgow, UK; 6 Department of Neurosurgery, Sheffield Teaching Hospitals Foundation Trust, Sheffield, UK; 7 Sussex Cancer Centre, University Hospitals Sussex NHS Foundation Trust, Brighton, UK; 8 Cambridge University Hospitals NHS Foundation Trust, Cambridge, UK; 9 National Hospital for Neurology and Neurosurgery, Brain Tumour Service (Neuro-Oncology), London, UK; 10 Department of Neurosurgery, University Hospitals Sussex NHS Foundation Trust, Brighton, UK; 11 Section of Neurosurgery, Department of Surgical Sciences, University of Otago, Dunedin School of Medicine, Dunedin, New Zealand; 12 Department of Biochemistry and Biomedicine, University of Sussex, Falmer, Brighton, UK

**Keywords:** Glioblastoma, Older adults, WHO CNS 5, CIMPACT-NOW, Survival outcomes

## Abstract

**Purpose:**

Glioblastoma is the most common primary brain cancer in adults, with half of cases diagnosed in patients aged ≥ 65 years. However, definitions of older adults vary, resulting in treatment variation between centres. This study aimed to report on the treatment intensity and survival outcomes in older patients with glioblastoma diagnosed according to WHO CNS 5 classification.

**Methods:**

We conducted a retrospective, multicentre cohort study (Histo-Mol GBM Collaborative) of consecutive patients with pathologically confirmed glioblastoma, IDH-wildtype diagnosed in 2021, according to the 2021 WHO CNS 5 classification, across 52 centres in the UK, Ireland, New Zealand, and Australia. Demographic, molecular, treatment, and survival data were analysed.

**Results:**

Of 1,857 patients, 863 (46.5%) were aged ≥ 65 years. Older patients had worse performance status, were more likely to have a biopsy, and received less intensive oncological therapy. Molecular testing was less comprehensive for older patients, but in those patients tested there was no difference with older age. Median overall survival declined with older age, with the steepest reduction in patients ≥ 70 years. Patients aged 65–69 derived comparable benefit from conventionally fractionated chemoradiation to adults aged < 65, whereas outcomes in those aged ≥ 70 were equivalent when treated with hypofractionated or conventionally fractionated chemoradiation. In multivariate analysis, oncological treatment intensity, completion of adjuvant therapy, and MGMT promoter methylation were independent predictors of survival.

**Conclusions:**

This international clinical dataset of adults diagnosed with glioblastoma since the introduction of the WHO CNS 5 criteria supports the use of conventionally fractionated chemoradiation in fit patients < 70, while hypofractionated regimens are appropriate for those ≥ 70. Surgical extent, treatment intensity, and MGMT methylation remain key determinants of survival in older patients with glioblastoma.

**Supplementary Information:**

The online version contains supplementary material available at 10.1007/s11060-026-05628-x.

## Introduction

Glioblastoma is the most common primary brain cancer in adults and the diagnosis of glioblastoma is highest amongst older adults. Incidence peaks between 65 and 79 years and over half of patients diagnosed with glioblastoma are aged over 65 [1, 2]. Older patients have poorer treatment outcomes: between the ages of 30 and 85, median survival reduces by 0.5–4.0 months for every year of age gained, and 5-year overall survival rates have been reported as only 0.2% for patients aged over 65, compared to 4% for all age groups [1, 3].

The definition of “older” patients with glioblastoma (oGBM) remains contentious. While age ≥ 65 is commonly associated with reduced treatment response and completion, a subset of patients still achieve outcomes comparable to younger adults when treated with more intensive therapies, and a systematic evaluation of outcomes for oGBM patients using different age thresholds treated according to contemporary clinical practice is lacking [4]. More holistic measures of “biological age” and frailty assessment, including cognitive or physical reserve, or brain volumetric analyses [5] may better identify candidates for maximal therapy, but chronological age thresholds are still used to direct molecular testing and treatment strategy [6–8].

Potential differences in underlying tumour biology and the impact of the 2021 revision of glioblastoma diagnostic criteria on molecular and histological features in oGBM remains unclear. Historically, older adults with glioblastoma were thought to display fewer distinct molecular alterations, and instead more commonly display molecular markers of poor prognosis [7]. However, these reports are collated prior to the revised 2021 Consortium to Inform Molecular and Practical Approaches to CNS Tumor Taxonomy (cIMPACT-NOW) [9] and World Health Organisation (WHO) Classification of Tumours of the Central Nervous System (CNS) 5 [7]. The revised schema includes both histological and molecular features, that are independently sufficient for a glioblastoma diagnosis and specifically excludes isocitrate dehydrogenase mutant (IDHmut) tumours that are more associated with younger adults [10]. Histopathological criteria for diagnosis of GBM (hGBM) includes necrosis and microvascular proliferation, while molecular criteria (mGBM) require: a telomerase reverse transcriptase promoter (*pTERT*) mutation, epidermal growth factor receptor (*EGFR*) amplification, or the combined gain of chromosome 7 and loss of chromosome 10 (chr 7+/10-). These criteria specifically exclude IDH-mutant tumours, which are more common in younger adults. This new mGBM diagnosis is made when histopathological criteria are not met.

This reclassification has implications for our understanding of the existing literature regarding molecular alterations in older adults, and whether changes in diagnostic classification have influenced clinical decision making and treatment responses for oGBM patients. Therefore, this manuscript addresses a clinical need to describe the characteristics and outcomes of a real-world cohort of older patients, whose diagnoses are informed by the most recent cIMPACT-NOW and WHO classifications.

The Histo-Mol GBM Collaborative is an international, multicentre retrospective real-world cohort study, including consecutive IDHwt glioblastoma patients diagnosed during a single year (2021) according to the WHO CNS 5 classification [11]. This cohort study provides individual patient level information regarding presentation, tumour treatment characteristics and survival outcomes for mGBM and hGBM patients, including how the 2021 diagnostic criteria contribute to our understanding of oGBM.

## Methods

### Study design and approval

This retrospective cohort study followed STROBE reporting guidelines [12]. Ethical approval was obtained from the University Hospitals Sussex (UHSussex) Clinical Outcomes and Effectiveness Committee (ref:1862). Institutional ethical review board approval was secured at each site. Additional ethical approvals were granted by the New Zealand Health and Disabilities Ethics Committee (ref:2024EXP21165) and the University of Otago Human Ethics Committee (Health) (ref: HD24/002). The study complied with the Declaration of Helsinki.

### Participants

Consecutive patients undergoing biopsy or resection between 1 January and 31 December 2021 and newly diagnosed with IDHwt glioblastoma, as per WHO CNS 5, were identified from 52 centres in the UK, Republic of Ireland, New Zealand, and Australia (Supplemental Table 1). Cases were identified and cross-referenced from neuropathology, neurosurgery, and neuro-oncology records. Patients who opted out of health record sharing were excluded. Diagnosis and treatment followed local protocols. Patients who did not receive a tissue diagnosis and/or received Best Supportive Care (BSC) with no surgical intervention were excluded.

### Data collection

Demographic, clinical, tumour, treatment and survival data were collected from medical records and managed using REDCap [13, 14]. Follow up period was 34 months. Older GBM (oGBM) are defined as patients aged ≥ 65 years of age. Age was stratified by < 65 years, 65–69 years, 70–74 years, 75–79 years, 80–84 years and ≥ 85 years. For stratified statistical analysis, data were analysed as both dichotomous groups (< 65/≥65 years) and stratified groups (< 65 years, 65–69 years, 70–74 years, 75–79 years, ≥ 80 years. *MGMT* promoter methylation was classified as unmethylated (0–10%) or methylated (> 10%) using primarily MGMT methylation array, but also pyrosequencing assay types [11, 15]. Surgery was recorded as biopsy or resection, which was subcategorised into gross total if ≥ 95%, or partial/subtotal if < 95% of contrast enhancing tumour was removed, as assessed and documented retrospectively from post-operative MRI imaging reports, neuro-oncology MDT (Multi-Disciplinary Team) decisions (which include surgical, radiological, oncological, histological and nursing representatives) and neurosurgical operation notes. Radiotherapy (RT) volumes were collected from Planned Treatment Volumes (PTV). Oncological therapy was categorised as: none, temozolomide alone, hypofractionated radiotherapy (typically 40 Gy/15#, EQD2 40–49 Gy), hypofractionated chemoradiotherapy, conventionally fractionated chemoradiotherapy (typically 60 Gy/30#, EQD2 53–61 Gy), or other (including palliative radiotherapy and conventionally fractionated radiotherapy alone). Concurrent therapy refers to TMZ (Temozolomide) treatment given at the same time as radiotherapy, while adjuvant therapy refers to additional TMZ treatment given following concurrent therapy, to maintain disease control. Treatment intensity was further grouped as surgery only, intermediate (oncological therapy listed previously, not meeting the criteria for aggressive), or aggressive (radiotherapy > 40 Gy, either 1.8-2 or > 2 Gy/fraction with concurrent chemotherapy). Short course chemoradiotherapy with > 40.05 Gy, the Perry et al. regimen, is included in the aggressive subgroup, in line with previous publications [1, 11, 16].

### Outcomes

Overall survival (OS) was measured from diagnostic surgery of glioblastoma to death or last follow-up. Progression-free survival (PFS) was measured from surgery to locally assessed radiological or clinical progression or censored at death/last follow-up.

### Statistical analysis

Analyses were performed using SPSS v29.0.2.0 (IBM, Chicago, IL) and R v4.3.0 (R Foundation, Vienna, Austria). Categorical variables were compared using Chi-squared with Cramér’s V effect size testing. Continuous variables were compared with two-way ANOVA with *post hoc* Tukey’s Honestly Significant Difference (THSD) and Bonferroni tests where required. Follow-up duration was calculated using the reverse Kaplan–Meier method. Overall and progression-free survival were estimated by Kaplan–Meier and compared with log-rank tests. Cox proportional hazards regression was used for univariate and multivariate analyses, with variables showing *p* < 0.01 on univariate analysis included in the multivariate model. Two-sided *p* < 0.05 was considered significant.

## Results

### Clinical Characteristics

Of the 1,857 patients with pathologically confirmed glioblastoma in 2021, 863 (46.5%) were aged ≥ 65 years (oGBM). Stratification by age demonstrated 994 patients (53.5%) aged < 65 years, 325 (17.5%) aged 65–69 years, 302 (16.3%) aged 70–74 years, 183 (9.9%) aged 75–79 years, and 53 (2.9%) aged ≥ 80 years. Of oGBM patients, 552 were male (64.0%) and 311 (36.0%) were female, with similar distributions (~ 60–70% male) identified in all age categories.

Older age group was significantly associated with poorer performance status (PS). Patients < 65 years most frequently presented with a performance status of 0–1 (84.7%), compared with 77.5% at 65–69, 71.2% at 70–74, and 70.3% at 75–79. This trend was statistically significant (*p* < 0.001). The proportion increased to 76.7% in those aged ≥ 80, likely reflecting selection bias, whereby only the fittest older patients underwent surgical intervention and tissue diagnosis, and were therefore included in the cohort. Conversely, higher PS scores (≥ 2) were more common in older patients, with PS 2–4 increasing from 15.4% in < 65 to approximately 22–24% in those ≥ 70.

Older patients were less likely to report seizures, anti-epileptic drug (AED) use and headaches compared to younger patients, but more likely to describe motor and speech symptoms (all *p* < 0.05, Chi-square analysis < 65 vs. ≥65 and five age categories, see *Methods* and Supplemental Tables 1 and 2). Difference in headache reporting with increasing age had the strongest effect size amongst recorded symptoms (0.2073, Cramér’s V). Of note, no significant associations between age group and gender, or age group and reported steroid use were observed.

**Fig. 1:**
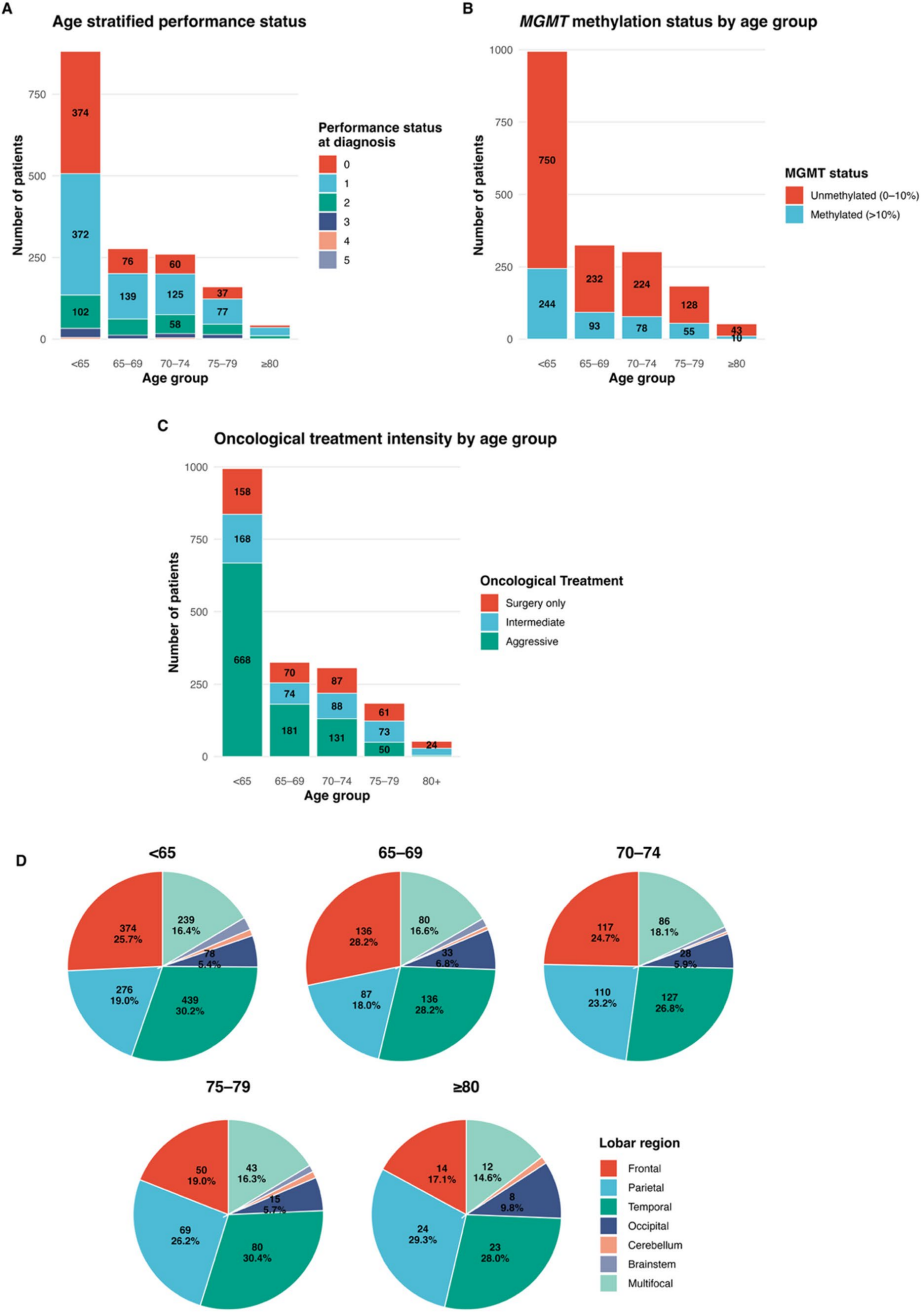
Overview of clinical characteristics** (A)** Stacked bar chart illustrating reported performance status (PS) for patients stratified by age category. **(B)** Stacked bar chart illustrating MGMT methylation status (< 10% unmethylated, ≥ 10% methylated) stratified by age group. **(C)** Stacked bar chart illustrating oncological treatment intensity (as defined by Le Calvez et al. 2025) stratified by age group. **(D)** Pie charts illustrating biopsy or resection location for patients stratified by age category

### Histological and anatomical characteristics

Older age was significantly associated with specific tumour locations. When comparing the < 65 and ≥ 65 age groups, there was a greater likelihood of parietal lobe involvement (*p* < 0.01) and reduced likelihood of brain stem involvement (*p* = 0.04). When comparing across all five age categories, there remained a greater likelihood of parietal lobe involvement (*p* < 0.01), but a reduced likelihood of frontal lobe involvement (*p* = 0.008). In contrast, no significant associations were observed with temporal, occipital or cerebellar involvement.

Older patients were less likely to undergo molecular testing for EGFR amplification, chr 7+/10- and pTERT mutation testing. These variants were tested in 7–15% fewer cases in the ≥ 65 vs. <65 age group (Supplemental Table 2). When comparing only individuals who received testing, there is no association between age and differing rates of these three variants (all *p* ≥ 0.155). Unlike other molecular testing, MGMT testing was comparable across age groups. MGMT promoter methylation status did not differ with increasing age (dichotomised as ≤ 10% or > 10% methylation coverage, Fig. 1C and Supplemental Tables 1 and 2).

### Treatment characteristics

Age group was significantly associated with surgical treatment strategy. Patients aged ≥ 65 were more likely to receive biopsy rather than resection (37.1% vs. 27.1%), and for those older patients who underwent resection, they were more likely to have a subtotal or partial (< 95%) resection (assessed locally on post-operative MRI) within 72 h of surgery, compared to patients aged < 65 years (*p* < 0.05).

Age group was significantly associated with oncological treatment strategy. Patients aged < 65 received significantly more intensive treatment than all older groups (*p* < 0.01). Older patients were more likely to receive reduced-intensity approaches, classified as surgery only or intermediate-intensity regimens, including hypofractionated radiotherapy, as reported previously [16].

Hypofractionated radiotherapy was almost exclusively used in older patients, with only two individuals < 65 receiving this reduced-intensity regimen. The most pronounced reduction in treatment intensity occurred in patients aged ≥ 70, while differences among the oldest groups (75–79 and ≥ 80) were smaller and non-significant, suggesting a plateau in treatment rates beyond this age. Most patients in the 65–69 age category did receive aggressive treatment, including both conventionally fractionated and hypofractionated chemoradiotherapy (Aggressive treatment, 181/325, 56%). However, this was a reduced proportion of the 65–69 age category as compared to the < 65 age group (Aggressive treatment 668/994, 67%), potentially reflecting the lower PS in the 65–69 age category (PS 0–1 215/277, 78%) compared to the < 65 age group (PS 0–1 746/881, 85%).

### Oncological treatment tolerance

Completion of post-surgical, oncological treatment is a pragmatic surrogate for treatment tolerance, reflecting patient fitness, resilience to toxicity and the ability to sustain multimodality therapy, while non-completion typically signals poor tolerance or decline in PS. Completion of concurrent chemoradiotherapy (87.2% vs. 87.0%) and adjuvant therapy (42.8% vs. 40.0%) were comparable between < 65 and ≥ 65 age groups. Concurrent treatment completion rates remained above 85% for all age categories, although absolute patient numbers were smaller in older age groups (Supplemental Table 2). When assessing adjuvant treatment completion across age categories, completion rates were similar in patients aged < 75 (~ 40–45%), but lower in those aged ≥ 75, with 11 (24.0%) of 75–79-year-olds and 4 (36.4%) of ≥ 80-year-olds completing therapy. Reasons for discontinuation differed by age. Progression was more common in older patients (84.0% vs. 74.5%), while non-haematological toxicity was more frequent in younger patients (17.2% vs. 8.0%). Haematological toxicity was slightly higher in older patients (8.0% vs. 6.0%). Overall, discontinuation due to toxicity was more common in younger patients (31.1% vs. 20.6%; *p* < 0.001).

Planned treatment volumes (PTV) was broadly comparable between age groups: the < 65 group had a mean PTV of 364 cc (median 336; range 96–1867) while the ≥ 65 group had a mean PTV of 361 cc (median 339; range 80–1300). PTV were similar among patients receiving conventionally fractionated CRT (means 365 vs. 370 cc, medians 336 vs. 353 cc), with a wider range in patients < 65 (44–1431 cc) compared to those ≥ 65 (117–780 cc). Concurrent or adjuvant treatment completion rates did not vary significantly by PTV within either age group (Supplemental Fig. 1, 2). This may reflect the comparable rates of concurrent and adjuvant therapy completion between age groups, and/or effective patient selection for treatment and supports results from a clinical trial setting [5].

### Treatment at disease recurrence

Confirmed progression (as defined by clinical consensus) after initial therapy was common across all ages within the data collection period (79.6% <65 vs. 70.3% ≥65). The likelihood of receiving subsequent treatment declined significantly with age: 52% of patients < 65 received second-line therapy compared with 31% ≥65 (*p* < 0.01), while 44% of patients aged < 65 received third-line therapy, compared with 33% ≥65 (*p* = 0.059). While treatment type (surgery, re-irradiation, systemic therapy, clinical trials) did not vary significantly for second-line therapy, there was some age-related variation in third-line choices when accessed across all age categories (*p* = 0.044). Most patients aged ≥ 65 years received systemic therapy (78.9% vs. 74.2%), with smaller proportions undergoing surgery (9.7% vs. 5.3%), re-irradiation (7.3% vs. 7.9%), or enrolment in clinical trials (3.2% vs. 0%), though interpretation is limited by small subgroup numbers. Confirmed progression rates after each line of therapy were consistent across age groups (≈ 60–70%) and not significantly associated with age. Fourth-line treatments were uncommon across all age groups. Across second-, third-, and fourth-line settings, systemic therapy options varied by age, with temozolomide and lomustine/CCNU predominating overall; younger patients (< 65) showed a more diverse treatment mix including PCV, bevacizumab, and combination therapies, whereas increasing age was associated with greater use of single-agent therapies, particularly lomustine/CCNU at later lines (Supplemental Fig. 3).

### Survival and treatment benefit

**Fig. 2:**
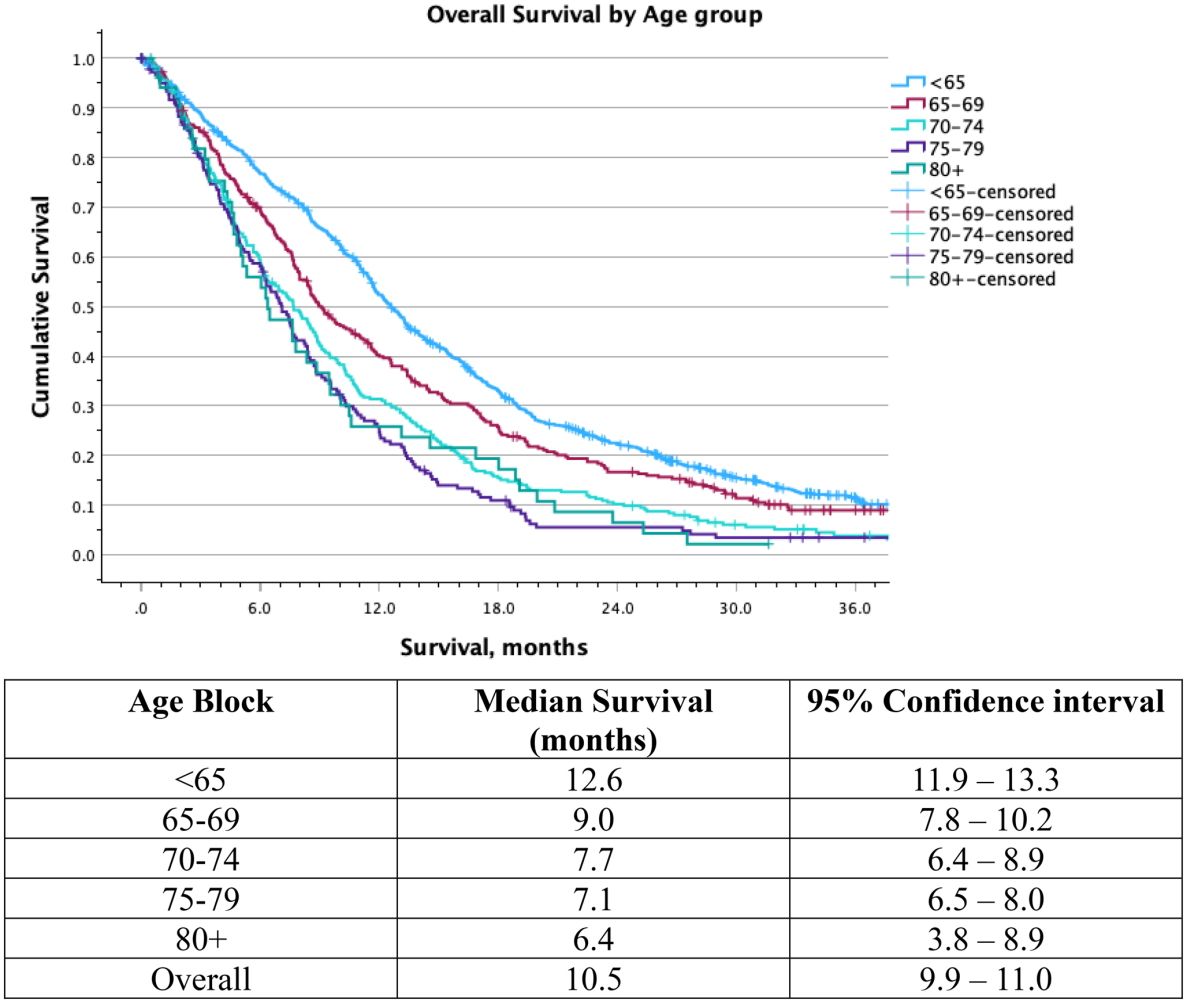
Overall survival by age group in patients with glioblastoma. Kaplan–Meier survival curves demonstrate progressively shorter overall survival with increasing age at diagnosis for all patients, regardless of treatment regimen.

Kaplan–Meier analysis demonstrated the well documented association between increasing age and reduced overall survival (Fig. 2). Patients aged < 65 years had the longest survival (median overall survival, mOS 12.6 months (11.9–13.3), with progressively worse outcomes seen in older age groups (65–69 years: mOS 9.0 months (7.8–10.2); 70–74 years: mOS 7.7 months (6.3–8.9); 75–79 years: 7.1 mOS months (6.1–8.0); ≥80 years: mOS 6.4 months (3.8–8.9)). The separation of curves was most pronounced within the first 12–18 months, after which survival rates converged at very low levels across all ages, regardless of treatment modality.

Survival varied markedly by treatment intensity across age categories (see Supplemental Table 4). Patients receiving no oncological therapy had the poorest outcomes, regardless of surgical intervention. Median survival was under 4 months, and no significant age effect was found. Median survival for patients who underwent biopsy was, as expected, lower than for those patients receiving resection (either subtotal or total), regardless of intensity of oncological therapy (Intermediate therapy: 6.6 (5.4–7.8) vs. 9.6 (8.6–10.5) months; Aggressive therapy: 11.1 (9.6–12.3) vs. 16.6 (15.7–17.5) months). The only treatment group for which age did make a significant difference to survival was resection followed by Intermediate-intensity oncological therapy. Among patients receiving intermediate-intensity treatment, the majority had unmethylated MGMT tumours across all age groups, including < 65 (77.4% unmethylated) and ≥ 65 (approximately 75–84% unmethylated across subgroups). The proportion of MGMT-methylated tumours remained relatively consistent across age categories (16–24%).

When restricting analysis to patients with good performance status (PS 0–1), survival differences between < 65 and 65–69 years remained minimal for both treatment strategies (Supplemental Table 6). However, outcomes in patients ≥ 70 years diverged. Those treated with the Stupp protocol experienced substantially shorter survival compared with younger patients (< 65 years: 16.9 (15.7–18.0) months; 65–69 years: 17.1 (13.9–20.3) months; ≥70 years: 13.0 (8.9–17.1) months, *p* = 0.030)). For patients treated with the Perry protocol, survival was longer for the oldest age category, even compared to other PS 0–1 patients (< 65 years: 9.8 (7.8–11.9) months; 65–69 years: 11.0 (7.0–15.0) months; ≥70 years 13.9 (12.1–15.7) months, *p* = 0.127)).

### Factors influencing overall survival in the 65–69 and ≥ 70 age groups

On univariate analysis, older age, poorer performance status, unmethylated MGMT, less extensive resection and less intensive oncological treatment were each significantly associated with shorter overall survival (age 65–69 vs. ≥70, *p* = 0.045, all else *p* < 0.001). Median survival declined from 9.0 months in patients aged 65–69 to 6.7 months in those ≥ 70 years, while survival improved stepwise with greater extent of resection (biopsy alone, < 95%, > 95%), see Table 1.

**Table 1:** Univariate and multivariate factors influencing overall survival in the 65-69 and ≥70 age groups

Univariate and multivariate results			
Variable	Comparison	Adjusted HR (95% CI)	Adjusted p value
Age group	65-59 vs. ≥70	1.008 (0.864-1.189	0.927
MGMT status	Methylated vs. unmethylated	0.601 (0.499-0.723)	<0.001
Extent of resection	Stepwise(biopsy < partial < total)	0.840 (0.752-0.938)	<0.001
PS	0-4	1.174 (1.067-1.291)	<0.001
Oncological treatment	Surgery only, Intermediate, Aggressive*	0.437(0.324-0.589)	<0.001

Univariate results from Kaplan-Meier (Log-rank test), multivariate results from Cox regression. CI: Confidence Interval, HR: Hazard Ratio; MGMT: O6-methylguanine methyltransferase; PS: performance status. *Oncological treatment intensity as defined by Le Calvez et al. 2025, see Methods

**Fig. 3:**
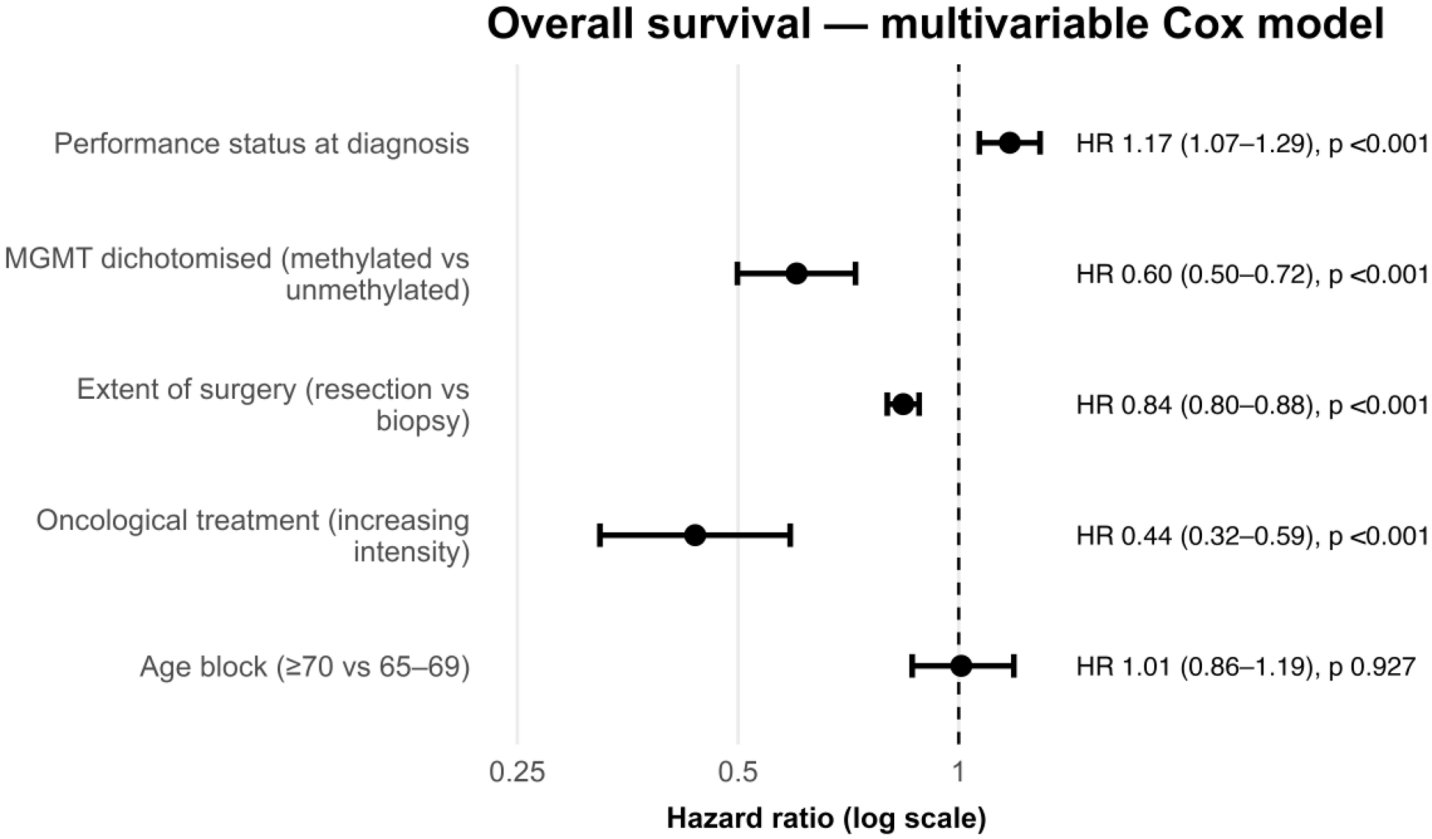
Multivariable Cox proportional hazards regression. Hazard ratios (HR) are shown on a logarithmic scale with 95% confidence intervals represented by horizontal bars

On multivariable Cox regression, several factors retained independent prognostic significance (Fig. 3). Increasing intensity of oncological treatment (HR 0.44, 95% CI 0.39–0.49, *p* < 0.001), MGMT promoter methylation (HR 0.60, 95% CI 0.50–0.72, *p* < 0.001) and extent of surgery (resection vs. biopsy, HR 0.840 (95% CI 0.752–0.938), *p* < 0.001) were associated with better outcomes, whilst worse performance status led to worse survival (HR 1.17, 95% CI 1.07–1.29, *p* < 0.001). However, when accounting for these factors there was no difference in survival for patients 65–70 or > 70 (HR 1.01, 95% CI 0.85–1.19, *p* = 0.927), in contrast to the univariate results.

## Discussion

In this large, international, real-world cohort of consecutive patients with IDH-wildtype glioblastoma diagnosed in 2021 according to WHO CNS 5, we provide the most comprehensive description of outcomes in older adults (≥ 65 years) in the context of updated histo-molecular classifications yet published. Our findings confirm the established association between increasing age and poorer outcomes, while highlighting the role of performance status and extent of resection as important co-factors.

In this cohort, we demonstrate lower rates of total resection in older patients, which likely reflect a clinical balance between maximising resection and minimising post-operative neurological morbidity, particularly in the context of frailty and reduced physiological reserve, which are recognised determinants of surgical decision-making and operative risk in glioblastoma [17–19].

We demonstrate that molecular variants EGFR amplification, chr 7+/10- chromosomal rearrangement and pTERT mutation were consistent between age groups, when tested for. The lack of testing among older adults reflects WHO CNS 5 classification guidance, which states that sequencing may not be needed in the setting of negative R132H IDH1 immunohistochemistry in patients > 55 years of age [6]. This may reflect more relative cases of histologically-confirmed GBM among oGBM patients, not requiring molecular testing to yield a diagnosis, although molecularly-confirmed GBM (i.e. the absence of histologically confirmed GBM) is not more associated with younger age in this cohort [11]. However, lack of molecular testing beyond immunohistochemistry characterisation limits older patents’ access to molecularly stratified treatments, especially in the context of clinical trials. *MGMT* promotor methylation was comparable between younger and older age groups, but at rates lower than in the literature (~ 26%, vs. 30–50% [20–23]), reported in studies which often describe smaller cohorts of patients, or patients in a clinical trial setting.

In this cohort, we demonstrate a greater likelihood of parietal lobe involvement, and reduced likelihood of brain stem involvement in older patients. The association between age and anatomical region could reflect a surgical selection bias among older patients, where tissue diagnosis (used to select this patient cohort) is biased towards cases where sampling is technically feasible with acceptable morbidity, rather than representing a predilection for oGBM to present in these anatomical regions (i.e. predilection for parietal sampling, but reduced brain stem sampling). However, if this were the case, we would expect both frontal and parietal lobe sampling to be higher among older adults, which is not the case in this cohort. There is also the possibility that this reflects a true difference in anatomical location, especially with relation to brain stem involvement, which is reported to be more common in younger patients with GBM IDHwt and the H3K27 variant (although the presence of this variant was not recorded for this cohort) [24].

Consistent with prior reports, older patients presented with worse baseline functional status, were more likely to undergo biopsy rather than resection and were less likely to receive aggressive oncological therapy (conventionally fractionated or hypofractionated concurrent chemoradiotherapy) compared to younger adults [1, 4, 25, 26]. Patients receiving intermediate therapy were more likely to have MGMT unmethylated tumours among both patients aged < 65 and ≥ 65 years. This reflects clinical decision making influenced by available data Perry et al. and Nordic trials which demonstrate limited benefit of temozolomide in MGMT-unmethylated glioblastoma [20, 27].

In this cohort, PTV were larger in patients receiving hypofractionated CRT compared with conventionally fractionated CRT, particularly in the ≥ 65 age groups, which may reflect the clinical decision to offer hypofractionated CRT when patients have a larger tumour, in order to reduce toxicity. Our finding that PTV volume was not associated with concurrent or adjuvant therapy completion rates supported earlier evidence from the BRITER clinical trial in which higher CSF to total intracranial volume ratio, but not PTV, was independently associated with worse overall survival [5].

In understanding the factors contributing to OS in the 65–69 and ≥ 70 age groups, univariate analyses demonstrated age, extent of surgery, intensity of oncological treatment, performance status, and MGMT promoter methylation to have significant associations individually, but there remained no survival difference between age categories 65–69 vs. ≥ 70 in multivariate analysis. In over 65-year-olds, age does not independently affect survival once other prognostic variables are accounted for, which include variables amenable to clinical decision making (e.g. extent of surgery, intensity of oncological treatment). When restricting analysis to patients with PS 0–1 who received aggressive therapy, outcomes for patients ≥ 70 years diverged by protocol: older patients treated with the Stupp protocol experienced substantially shorter survival compared with younger patients, while patients treated with the Perry protocol, survival was longer for the oldest age category. This may reflect other clinical parameters, beyond PS, which have resulted in patients aged < 65 being selected to receive a less intensive regimen. Bias towards treating older adults based on age alone, could alter surgical and therapeutic decision making, resulting in suboptimal resection or lower intensity therapies for older patients. This highlights the need for personalised approaches taking into account patients’ wishes, but also age specific biomarkers of treatment response, whether that be biological or functional (for example frailty assessments, comorbidity, prehabilitation or response to steroids) rather than chronological age alone [4, 28]. It also raises the question of the surgical and therapeutic approaches at different surgical units and treating centres, and whether rates of intervention at older age reflect differences in MDT decision making.

Our study has several strengths, including its large, multicentre design, real-world setting including all consecutively diagnosed patients, and contemporary nature, classified by the most recent WHO CNS 5 criteria. Limitations include the retrospective design, potential selection bias for pathological confirmation (especially in patients ≥ 80 years, who represent only the fittest subset undergoing biopsy/resection), lack of central review for radiology and radiotherapy planning, dosing or toxicity attribution and the availability of data (especially planning target volume) for some patients. Nevertheless, the cohort provides real world evidence to guide clinical decision-making in older adults with glioblastoma.

In summary, our study provides the largest contemporary resource consistent with the current diagnostic classification for counselling older patients with glioblastoma. Results support the continued use of the Stupp regimen in fit patients under 70, while hypofractionated chemoradiation was demonstrated to be an effective standard treatment for patients ≥ 70 years in this cohort. Performance status, treatment intensity, and MGMT promoter methylation status are key determinants of survival, reaffirming the importance of integrated histo-molecular classification and individualized treatment planning in older glioblastoma patients.

## Supplementary Information

Below is the link to the electronic supplementary material.

Supplementary Material 1

## Data Availability

The data underlying this study are available from the corresponding authors upon reasonable request and are accessible through the Health Data Gateway (Data Custodian ID: 123).

## **Appendix 1.** Histo-Mol GBM Collaborative Contributors:

**The Beatson West of Scotland Cancer Centre:** Attika Chaudhary, Sean Farrell, Simon Lammy.

**University Hospitals Birmingham NHS Foundation Trust:** Reshma David, Neha Rathod, Vinton Cheng.

**Newcastle upon Tyne Hospitals NHS Foundation Trust:** Alex Byers, Noah Shi Jie Lee, Ian Coulter.

**Leeds Teaching Hospitals NHS Trust:** Michael Clark, Paul Roberts, Christopher Hooper, Kavi Fatania, Stuart Currie.

**Auckland City Hospital:** Jayne Sheridan, Malinda Lucas, Davina McAllister, Peter Heppner.

**Clatterbridge Cancer Centre:** Jillian Sokratous, Marcus Rathbone, Shaveta Mehta.

**Kent Oncology Centre:** Katherine Ryan, Louisa Black, Samantha Forner.

**Nottingham University Hospitals NHS Trust:** Ia Robert-Montaner, Hawawu Muazu, Paul McDonnell, Daniele Scotto, Mihir Kocherlakota, Jake Hewitt, Joon Ho, Sangary Kathirgamakathigeyan.

**Cambridge University Hospitals NHS Foundation Trust:** Emily Lachmann, Rebecca Sen, Mareike Thompson.

**National Hospital for Neurology and Neurosurgery:** Adrianna Wong, Henry Ajah, Bernadette Kovacs, Ciaran Hill.

**Weston Park Cancer Hospital, Sheffield Teaching Hospitals:** Lalit Srimat Tirumala, Priyanka Augustine, Dominic Van Loan, Rebecca Anim-Boadu, Zephton Wedderburn, Ola Rominyi, Sophie T Williams.

**Hull University Teaching Hospitals NHS Trust:** Nathaniel Luke Hatton, Rebecca Hatton, Nur Aktar, Sanjay Dixit.

**Beaumont Hospital:** Suzanne Murphy, Gerard Lavelle, Mohammed Abdelsadig, Kate Connor, Yvonne Kirwan, Eloise Cowie, Riya Sharma.

**The Christie NHS Foundation Trust:** Marianna Theodoulou, Karan Patel.

**Lancashire Teaching Hospitals NHS Foundation Trust:** Christopher Chan, Luke McGurk, Benjamin Sanderson.

**Queen’s Hospital:** Amir Ameen, Alireza Shoakazemi, Louise Dulley.

**Sussex Cancer Centre:** Haddon Lathangue, Stephen David Robinson, Nektarios K Mazarakis, Cressida Lorimer, Edward Chandy, Giles Critchley.

**Wellington Hospital:** Michael Nichols, Andrew Parker.

**University Hospital Southampton NHS Foundation Trust:** Soham Bandyopadhyay, Carlo Lori, Sean Main, Jeng Ching.

**Mid and South Essex NHS Foundation Trust:** Femi Adeoye, George Sioftanos.

**Mount Vernon Cancer Centre:** Sabihya Wontumi, Swarna Arumugam, Thomas Carter.

**The Northern Ireland Cancer Centre:** David Conkey, Jacqui Harney.

**University Hospitals Bristol NHS Foundation Trust:** Lindsay Rayner, Lorna Hawley.Waikato Hospital: Sung-Min Jun, Heta Leinonen.

**The Royal Marsden Hospital NHS Foundation Trust:** James de Boisanger, Babushka Kalra, Antonia Creak, Liam Welsh.

**Cork University Hospital:** Lena Dablouk, Chantalle Berkhout, Jack Gleeson.

**Christchurch Hospital:** Benjamin Harley, Simon John.

**University Hospitals of Leicester NHS Trust:** Anupama Vjay, Shradha Bhagani.

**Royal Stoke University Hospital:** Adam Fullagar, Sumera Butt.

**Gloucestershire Hospitals NHS Foundation Trust:** Praveen T Tikiri Durage, Samir Guglani.

**University Hospitals Plymouth NHS Trust:** Lucy Counsell, Ellie Eddlmann, Elizabeth Lim.

**North Wales Cancer Centre:** Mohammed Hatata, Heba Shebani, Iffat Jurfa, Mazin Sirelkhatim.

**Portsmouth Hospitals University NHS Trust:** Elizabeth Wilson, Eleni Simpson.

**Norfolk and Norwich University Hospitals NHS Foundation Trust:** Andrew Ho.

**University Hospitals Dorset NHS Foundation Trust:** Jason Bowie, Mark Noble.

**University Hospitals of Derby and Burton NHS Foundation Trust:** Katherine Winfield, Vishnu Pillai, Mike Smith-Howell.

**Barts Health NHS Trust:** Paolo de Luna, Timothy Hill, Pavlos Christodoulou, Rachel Lewis.

**Royal Surrey Cancer Centre:** Rachel Macarthur, May Teoh.

**Imperial College Healthcare NHS Trust: **Mahbuba Choudhury, Miranda Bowman, Camilla Cavilli, Matthew Williams.

**NHS Grampian:** Adenike Williams, Rafael Moleron.

T**orbay and South Devon NHS Foundation Trust:** Timothy Norris.

**Velindre University NHS Trust:** Jennifer Golten, Tasia Aghadiuno, Jillian MacLean, James Powell.

**Royal Devon University Healthcare NHS Foundation Trust:** Sarah Kingdon, Anne McCormack.

**East Suffolk and North Essex NHS Foundation Trust:** Natalie Wheatley, Diana Lobo, Leila Bidwell, Jennifer Collins.

**James Cook Hospital:** Kohgulakuhan Yogalingam, Nick Wadd.

**Royal Cornwall Hospitals NHS Trust:** Daniel Duffy, Grant Stewart.

**NHS Tayside:** Sue Een Lau, Hannah Lord.

**Swansea Bay University Health Board:** Jennifer Kahan, Prashanth Bhat.

**United Lincolnshire Hospitals NHS Trust:** Gbenro Olukiran, Laura Walsh, Aurora Sanz-Torres.

**Royal Berkshire NHS Foundation Trust:** Ed Morgan Ruth Davis.

**Dunedin Hospital:** Michael Heckelmann, Ahmad Taha, Giles Critchley.

**Royal Prince Alfred Hospital:** Laveniya Satgunaseelan, Kimberley L Alexander.

## References

[1] Brodbelt A, Greenberg D, Winters T et al (2015) Glioblastoma in England: 2007–2011. Eur J Cancer 51:533–542. 10.1016/j.ejca.2014.12.014 25661102 10.1016/j.ejca.2014.12.014

[2] Ostrom QT, Cioffi G, Waite K et al (2021) CBTRUS Statistical Report: Primary Brain and Other Central Nervous System Tumors Diagnosed in the United States in 2014–2018. Neurooncology 23:iii1–iii105. 10.1093/neuonc/noab200 10.1093/neuonc/noab200 PMC8491279 34608945

[3] Poon MTC, Sudlow CLM, Figueroa JD, Brennan PM (2020) Longer-term (≥ 2 years) survival in patients with glioblastoma in population-based studies pre- and post-2005: a systematic review and meta-analysis. Sci Rep 10:1–10. 10.1038/s41598-020-68011-4 32669604 10.1038/s41598-020-68011-4 PMC7363854

[4] Mazarakis NK, Robinson SD, Sinha P et al (2024) Management of glioblastoma in elderly patients: A review of the literature. Clin Translational Radiation Oncol 46:100761. 10.1016/j.ctro.2024.100761 10.1016/j.ctro.2024.100761 PMC10945210 38500668

[5] Lorimer C, Mills S, Chalmers A et al (2024) Baseline total brain volume predicts changes in quality of life and overall survival after cranial radiotherapy in older patients with glioblastoma: Results from the prospective BRITER study. Neuro-Oncology Pract 11:413–420. 10.1093/nop/npae019 10.1093/nop/npae019 PMC11241370 39006523

[6] Louis DN, Perry A, Reifenberger G et al (2016) The 2016 World Health Organization Classification of Tumors of the Central Nervous System: a summary. Acta Neuropathol 131:803–820. 10.1007/s00401-016-1545-1 27157931 10.1007/s00401-016-1545-1

[7] Louis DN, Perry A, Wesseling P et al (2021) The 2021 WHO Classification of Tumors of the Central Nervous System: Clinical implications. Neurooncology 23:1231–1251. 10.1093/neuonc/noab106 10.1093/neuonc/noab106 PMC8328013 34185076

[8] Weller J, Katzendobler S, Niedermeyer S et al (2023) Treatment benefit in patients aged 80 years or older with biopsy-proven and non-resected glioblastoma is dependent on MGMT promoter methylation status. J Neurooncol 163:407–415. 10.1007/s11060-023-04362-y 37289281 10.1007/s11060-023-04362-y PMC10322768

[9] Brat DJ, Aldape K, Colman H et al (2018) cIMPACT-NOW update 3: recommended diagnostic criteria for Diffuse astrocytic glioma, IDH-wildtype, with molecular features of glioblastoma, WHO grade IV. Acta Neuropathol 136:805–810. 10.1007/s00401-018-1913-0 30259105 10.1007/s00401-018-1913-0 PMC6204285

[10] Miller JJ, Gonzalez Castro LN, McBrayer S et al (2023) Isocitrate dehydrogenase (IDH) mutant gliomas: A Society for Neuro-Oncology (SNO) consensus review on diagnosis, management, and future directions. Neurooncology 25:4–25. 10.1093/neuonc/noac207 10.1093/neuonc/noac207 PMC9825337 36239925

[11] Robinson SD, Kingdon S, Williams ST et al (2026) Understanding the difference in symptoms and outcomes between glioblastoma patients diagnosed based on histological or molecular criteria: a retrospective cohort analysis from the Histo-Mol GBM collaborative. J Neurooncol 176:157. 10.1007/s11060-025-05364-8 41504931 10.1007/s11060-025-05364-8 PMC12783167

[12] von Elm E, Altman DG, Egger M et al (2008) The Strengthening the Reporting of Observational Studies in Epidemiology (STROBE) statement: guidelines for reporting observational studies. J Clin Epidemiol 61:344–349. 10.1016/j.jclinepi.2007.11.008 18313558 10.1016/j.jclinepi.2007.11.008

[13] Harris PA, Taylor R, Thielke R et al (2009) Research electronic data capture (REDCap)—A metadata-driven methodology and workflow process for providing translational research informatics support. J Biomed Inform 42:377–381. 10.1016/j.jbi.2008.08.010 18929686 10.1016/j.jbi.2008.08.010 PMC2700030

[14] Harris PA, Taylor R, Minor BL et al (2019) The REDCap consortium: Building an international community of software platform partners. J Biomed Inform 95:103208. 10.1016/j.jbi.2019.103208 31078660 10.1016/j.jbi.2019.103208 PMC7254481

[15] McAleenan A, Kelly C, Spiga F et al (2021) Prognostic value of test(s) for O6-methylguanine–DNA methyltransferase (MGMT) promoter methylation for predicting overall survival in people with glioblastoma treated with temozolomide. Cochrane Database Syst Reviews. 10.1002/14651858.CD013316.pub2 10.1002/14651858.CD013316.pub2 PMC8078495 33710615

[16] Le Calvez K, Mauricaite R, Treasure P et al (2025) Adult glioblastoma in England: Incidence, treatment, and outcomes with novel population-based strata. Cancer Epidemiol 97:102811. 10.1016/j.canep.2025.102811 40203511 10.1016/j.canep.2025.102811

[17] Brown TJ, Brennan MC, Li M et al (2016) Association of the Extent of Resection With Survival in Glioblastoma: A Systematic Review and Meta-analysis. JAMA Oncol 2:1460. 10.1001/jamaoncol.2016.1373 27310651 10.1001/jamaoncol.2016.1373 PMC6438173

[18] Chaichana KL, Chaichana KK, Olivi A et al (2011) Surgical outcomes for older patients with glioblastoma multiforme: preoperative factors associated with decreased survival. Clinical article. J Neurosurg 114:587–594. 10.3171/2010.8.JNS1081 20887095 10.3171/2010.8.JNS1081 PMC4020429

[19] Cloney M, D’Amico R, Lebovic J et al (2016) Frailty in Geriatric Glioblastoma Patients: A Predictor of Operative Morbidity and Outcome. World Neurosurg 89:362–367. 10.1016/j.wneu.2015.12.096 26775233 10.1016/j.wneu.2015.12.096

[20] Malmström A, Grønberg BH, Marosi C et al (2012) Temozolomide versus standard 6-week radiotherapy versus hypofractionated radiotherapy in patients older than 60 years with glioblastoma: the Nordic randomised, phase 3 trial. Lancet Oncol 13:916–926. 10.1016/S1470-2045(12)70265-6 22877848 10.1016/S1470-2045(12)70265-6

[21] Mak KS, Agarwal A, Qureshi MM, Truong MT (2017) Hypofractionated short-course radiotherapy in elderly patients with glioblastoma multiforme: an analysis of the National Cancer Database. Cancer Med 6:1192–1200. 10.1002/cam4.1070 28440040 10.1002/cam4.1070 PMC5463088

[22] Lapointe S, Perry A, Butowski NA (2018) Primary brain tumours in adults. Lancet 392:432–446. 10.1016/S0140-6736(18)30990-5 30060998 10.1016/S0140-6736(18)30990-5

[23] Weller M, Stupp R, Reifenberger G et al (2010) MGMT promoter methylation in malignant gliomas: ready for personalized medicine? Nat Reviews Neurol 6:39–51. 10.1038/nrneurol.2009.197 10.1038/nrneurol.2009.197 19997073

[24] Saratsis AM, Knowles T, Petrovic A, Nazarian J (2024) H3K27M mutant glioma: Disease definition and biological underpinnings. Neurooncology 26:S92–S100. 10.1093/neuonc/noad164 10.1093/neuonc/noad164 PMC11066930 37818718

[25] Iwamoto FM, Reiner AS, Panageas KS et al (2008) Patterns of care in elderly glioblastoma patients. Ann Neurol 64:628–634. 10.1002/ana.21521 19107984 10.1002/ana.21521

[26] Gulati S, Jakola AS, Johannesen TB, Solheim O (2012) Survival and Treatment Patterns of Glioblastoma in the Elderly: A Population-Based Study. World Neurosurg 78:518–526. 10.1016/j.wneu.2011.12.008 22381305 10.1016/j.wneu.2011.12.008

[27] Perry JR, Laperriere N, O’Callaghan CJ et al (2017) Short-Course Radiation plus Temozolomide in Elderly Patients with Glioblastoma. N Engl J Med 376:1027–1037. 10.1056/nejmoa1611977 28296618 10.1056/NEJMoa1611977

[28] Lorimer CF, Walsh G, MacKinnon M, et al (2019) Geriatric assessment of glioblastoma patients is feasible and may provide useful prognostic information. Neurooncol Pract 6(6):456–464. 10.1093/nop/npz040 10.1093/nop/npz040 PMC7318852 32626586

