# Supplementary material for: Report of treatment intensity and survival outcomes in older patients with glioblastoma diagnosed according to WHO CNS 5 classification
Source: J Neurooncol. 2026 Jun 4;178(2):41. doi: 10.1007/s11060-026-05628-x (PMC13236755; doi:10.1007/s11060-026-05628-x)
Supplement: Supplementary file 1 — Supplementary Material 1 [file 11060_2026_5628_MOESM1_ESM.docx]

**Supplemental Table 1:** Patients Included by Country.

**Supplemental Table 2:** Clinical Characteristics by Age Dichotomised <65 vs. ≥65 years.

**Supplemental Table 3:** Clinical Characteristics by Age Groups.

**Supplemental Figure 1:** Concurrent therapy treatment termination due to toxicity and mean PTV compared between age categories <65 and ≥65 years.

**Supplemental Figure 2:** Adjuvant therapy treatment termination due to toxicity and mean PTV compared between age categories <65 and ≥65 years.

**Supplemental Table 4:** Survival data stratified by treatment regimen and age.

**Supplemental Table 5:** Survival data stratified by treatment for patients receiving hypofractionated chemoradiotherapy or conventionally fractionated chemoradiotherapy for different age groups (<65, 65-69 and ≥70 years of age) and all performance status groups (0-4).

**Supplemental Table 6:** Survival data stratified by treatment for patients receiving hypofractionated chemoradiotherapy or conventionally fractionated chemoradiotherapy for different age groups (<65, 65-69 and ≥70 years of age) and performance status groups 0-1.

Supplemental Table 1: **Patients Included by Country**

|  | Country | Patients (n) | Percent of total |
| --- | --- | --- | --- |
|  | England | 1,343 | 72.3% |
|  | New Zealand | 184 | 9.9% |
|  | Scotland | 127 | 6.8% |
|  | Republic of Ireland | 84 | 4.5% |
|  | Wales | 50 | 2.7% |
|  | Northern Ireland | 40 | 2.2% |
|  | Australia (Sydney) | 29 | 1.6% |
| Total | — | 1,857 | — |
| Percentages represent proportion of total sample (N = 1,857). | | |  |

Supplemental Table 2: **Clinical Characteristics by Age Dichotomised <65 vs. ≥65 years.**

| **Variable** | **Subcategory** | **<65 y** | **≥65 y** | $\boldsymbol{\chi}\boldsymbol{2}$  **(Cramer’s V)** |
| --- | --- | --- | --- | --- |
| Gender | Recorded | 994 | 863 |  |
|  | Male | 629  (63.3%) | 552  (64.0%) | 0.7973 |
|  | Female | 365  (36.7%) | 311  (36.0%) |  |
| Performance status^1^ | Recorded | 881 | 740 |  |
|  | 0 | 374  (42.5%) | 180  (24.5%) | **<0.001**  (0.2027) |
|  | 1 | 372  (42.2%) | 367  (51.1%) |  |
|  | 2 | 102  (11.6%) | 149  (20.2%) |  |
|  | 3 | 28  (3.2%) | 37  (5.0%) |  |
|  | 4 | 5  (0.6%) | 7  (0.9%) |  |
| Presenting complaint reported^2^ | Recorded | 994 | 863 |  |
|  | Seizures | 289  (29.1%) | 199  (23.1%) | **<0.001**  (0.06816) |
|  | Motor | 318  (32.0%) | 370  (42.9%) | **<0.001**  (0.1124) |
|  | Speech | 251  (25.3%) | 273  (31.6%) | **0.0027**  (0.07073) |
|  | Cognition | 239  (24.0%) | 255  (29.6%) | **<0.001**  (0.06212) |
|  | Behaviour | 70  (7.0%) | 70  (8.1%) | 0.4342 |
|  | Vision | 106  (10.7%) | 68  (7.8%) | **0.0484**  (0.04766) |
|  | Headache | 422  (42.5%) | 202  (23.4%) | **<0.001**  (0.2011) |
|  | Sensory | 116  (11.7%) | 94  (10.8%) | 0.6495 |
|  | GCS^2^ | 44  (4.4%) | 33  (3.8%) | 0.594 |
|  | Incidental | 14  (1.4%) | 14  (1.6%) | 0.8523 |
|  | Other | 139  (14.0%) | 114  (13.2%) | 0.6765 |
| Steroid use | Recorded | 972 | 844 |  |
|  |  | 774  (79.6%) | 688  (81.5%) | 0.3406 |
| AED3 use | Recorded | 986 | 857 |  |
|  |  | 476  (48.3%) | 355  (41.4%) | **0.00371**  (0.06869) |
| **Tumour location on MRI** | | | | |
| Tumour location | Recorded | 994 | 863 |  |
|  | Frontal | 374  (37.6%) | 317  (36.7%) | 0.727 |
|  | Parietal | 276  (27.8%) | 290  (33.6%) | **0.007476**  (0.06324) |
|  | Temporal | 439  (44.2%) | 366  (38.9%) | 0.4751 |
|  | Occipital | 78  (7.2%) | 84  (9.7%) | 0.1756 |
|  | Cerebellum | 16  (1.6%) | 9  (1.0%) | 0.3925 |
|  | Brainstem | 32  (3.2%) | 14  (1.6%) | **0.03951**  (0.05125) |
|  | Multifocal | 239  (24.1%) | 221  (25.6%) | 0.4686 |
| **Molecular diagnostics** | | | | |
| MGMT^4^ | Reported | 994 | 863 |  |
|  | MGMT  Methylation ≥10% | 244  (24.5%) | 236  (27.3%) | 0.1865 |
| EGFR^5^ | Reported | 956 | 850 |  |
|  | EGFR amplified | 166  (17.4%) | 108  (12.5%) | **<0.001**  (0.1119) |
|  | EGFR not amplified | 273  (28.6%) | 189  (21.9%) |  |
|  | EGFR not tested | 517  (54.1%) | 553  (64.1%) |  |
| Chr 7+/Chr 10- | Reported | 947 | 849 |  |
|  | Chr 7+/Chr 10- present | 75  (7.9%) | 33  (3.9%) | **<0.001**  (0.105) |
|  | Chr 7+/Chr 10-  absent | 118  (12.5%) | 77  (9.1%) |  |
|  | Chr 7+/Chr 10-  not tested | 754  (79.6%) | 739  (87.0%) |  |
| TERT^6^-mutation | Reported | 972 | 855 | **<0.001**  (0.1591) |
|  | TERT-mutation present | 370  (38.1%) | 226  (26.4%) |  |
|  | TERT-mutation  not present | 95  (9.8%) | 50  (5.8%) |  |
|  | TERT-mutation  not tested | 507  (52.2%) | 579  (67.7%) |  |
| Treatment received | | | | |
| Surgical treatment | Surgery reported | 994 | 863 |  |
|  | Biopsy | 269  (27.1%) | 320  (37.1%) | **<0.001**  (0.1074) |
|  | Resection | 725  (72.9%) | 543  (62.9%) |  |
| Extent of resection | Biopsy | 269  (27.1%) | 320  (37.1%) | **<0.001**  (0.1262) |
|  | Resection <95% | 324  (32.6%) | 287  (33.3%) |  |
|  | Resection ≥95% | 401  (40.3%) | 256  (29.7%) |  |
| Oncological treatment by intensity | Reported | 994 | 868 |  |
|  | Surgery alone | 158  (15.9%) | 242  (27.9%) | **<0.001**  (0.2513) |
|  | Intermediate | 168  (16.9%) | 260  (30.0%) |  |
|  | Aggressive | 668  (67.2%) | 366  (42.1%) |  |
| Firstline oncological treatment by regimen | Treatment reported | 749 | 577 |  |
|  | Temozolomide alone | 24  (2.6%) | 26  (4.5%) | **<0.001**  (0.4173) |
|  | Hypofractionated RT | 57  (6.3%) | 130  (22.5%) |  |
|  | Hypofractionated CRT  (Perry *et al.*) | 58  (6.4%) | 179  (31.0%) |  |
|  | Conventionally fractionated CRT  (Stupp *et al.*) | 610  (67.4%) | 242  (41.2%) |  |
| **Treatment completion** | | | | |
| Concurrent treatment | Reported | 682 | 385 |  |
|  | Completed | 595  (87.2%) | 335  (87.0%) | 0.9898 |
|  | Not completed | 87  (12.8%) | 50  (13.0%) |  |
| Adjuvant treatment | Reported | 575 | 317 |  |
|  | Completed | 246  (42.8%) | 127  (40.0%) | 0.4732 |
|  | Not completed | 329  (57.2%) | 190  (60%) |  |
| Adjuvant discontinuation reason | Reported | 337 | 149 |  |
|  | Toxicity  (haematological) | 20  (6.0%) | 12  (8.0%) | **0.02053**  (0.1275) |
|  | Toxicity  (non-haematological) | 58  (17.2%) | 12  (8.0%) |  |
|  | Progression | 251  (74.5%) | 125  (84.0%) |  |
| Stopped due to toxicity | Reported | 994 | 863 |  |
|  | Yes | 309  (31.1%) | 178  (20.6%) | **<0.001**  (0.1186) |
|  | No | 685  (68.9%) | 685  (79.4%) |  |
| **Additional Treatments** | | | | |
| 2nd Line Treatment | No | 373  (47.9%) | 406  (69.0%) | **<0.001**  (0.2117) |
|  | Yes | 406  (52.10%) | 182  (31.0%) |  |
| 2nd Line Treatment Type | Surgery | 85  (21.1%) | 36  (19.9%) | 0.90 |
|  | Radiotherapy  (no PTV overlap) | 7  (1.7%) | 6  (3.3%) |  |
|  | Radiotherapy (marginal PTV overlap) | 10  (2.5%) | 5  (2.8%) |  |
|  | Radiotherapy (reirradiation/full PTV overlap) | 13  (3.2%) | 5  (2.8%) |  |
|  | Systemic therapy | 279  (69.2%) | 125  (69.1%) |  |
|  | Clinical trial | 9  (2.2%) | 4  (2.2%) |  |
| 3rd Line Treatment | No | 158  (56.0%) | 77  (67.0%) | 0.059 |
|  | Yes | 124  (44.0%) | 38  (33.0%) |  |
| 3rd Line Treatment Type | Surgery | 12  (9.7%) | 2  (5.3%) | 0.814 |
|  | Radiotherapy  (no PTV overlap) | 5  (4.0%) | 2  (5.3%) |  |
|  | Radiotherapy (marginal PTV overlap) | 2  (1.6%) | 1  (2.6%) |  |
|  | Radiotherapy (reirradiation/full PTV overlap) | 9  (7.3%) | 3  (7.9%) |  |
|  | Systemic therapy | 92  (74.2%) | 30  (78.9%) |  |
|  | Clinical trial | 4  (3.2%) | 0 |  |
| 4th Line Treatment | No | 43  (58.1%) | 14  (60.9%) | 1.00 |
|  | Yes | 31  (41.9%) | 9  (39.1%) |  |
| 4th Line Treatment Type | Surgery | 3  (9.7%) | 0 | NA |
|  | Radiotherapy  (no PTV overlap) | 0 | 0 |  |
|  | Radiotherapy (marginal PTV overlap) | 1  (3.2%) | 0 |  |
|  | Radiotherapy (reirradiation/full PTV overlap) | 3  (9.7%) | 1  (11.1%) |  |
|  | Systemic therapy | 24  (77.4%) | 8  (88.9%) |  |
|  | Clinical trial | 0 | 0 |  |

Percentages represent proportion of patients with reported variable. ^1^Performance status documented at diagnosis. ^2^Presenting complaint dichotomized; ^3^GCS Glasgow Coma Score; ^4^AED Antiepileptic Drug; ^5^*MGMT* O^6^-methylguanine-DNA methyltransferase status dichotomized as <10%, ≥10%; ^6^*EGFR* Epidermal Growth Factor Receptor gene; ^7^*TERT* Telomerase Reverse Transcriptase gene; ^8^Chemoradiotherapy; ^9^PTV Planned Treatment Volume.

Supplemental Table 3: **Clinical Characteristics by Age Groups.**

| **Variable** | **Subcategory** | **<65 y** | **65<69 y** | **70<74 y** | **75<79 y** | **80+ y** | $\boldsymbol{\chi}\boldsymbol{2}$ **p value**  **(Cramer’s V)** |
| --- | --- | --- | --- | --- | --- | --- | --- |
| Gender | Recorded | 994 | 325 | 302 | 183 | 53 |  |
|  | Male | 629 (63.3%) | 196  (60.3%) | 206  (68.2%) | 113  (61.7%) | 37  (69.8%) | 0.2402 |
|  | Female | 365  (36.7%) | 129  (39.7%) | 96  (31.8%) | 70  (38.3%) | 16  (30.2%) |  |
| Performance status^1^ | Recorded | 881 | 277 | 260 | 155 | 43 |  |
|  | 0 | 374 (42.5%) | 76  (27.4%) | 60  (23.1%) | 37 (20.6%) | 7  (16.3%) | **<0.001**  (0.1101) |
|  | 1 | 372 (42.2%) | 139 (50.2%) | 125 (48.1%) | 77 (49.7%) | 26  (60.5%) |  |
|  | 2 | 102 (11.6%) | 50  (18.1%) | 58  (22.3%) | 32 (20.6%) | 9  (20.9%) |  |
|  | 3 | 28  (3.2%) | 11  4.0%) | 13  (5.0%) | 12  (7.7%) | 1  (2.3%) |  |
|  | 4 | 5  (0.6%) | 1  (0.4%) | 4  (1.5%) | 2  (1.3%) | 0  (0.0%) |  |
| **Presenting complaint** | | | | | | | |
| Presenting complaint reported^2^ | Recorded | 994 | 325 | 302 | 183 | 53 |  |
|  | Seizures | 289 (29.1%) | 84  (25.8%) | 67  (22.2%) | 41 (22.4%) | 7  (13.2%) | **0.01259**  (0.08285) |
|  | Motor | 318 (32.0%) | 137 (42.2%) | 123 (40.7%) | 86 (47.0%) | 24 (45.3%) | **<0.001**  (0.1174) |
|  | Speech | 251 (25.3%) | 86  (26.5%) | 116 (38.4%) | 58 (31.7%) | 13  (24.5%) | **<0.001**  (0.1082) |
|  | Cognition | 239 (24.0%) | 95  (29.2%) | 95  (31.5%) | 53 (29.0%) | 12  (22.6%) | 0.05989 |
|  | Behaviour | 70  (7.0%) | 30  (9.2%) | 23  (7.3%) | 13  (7.1%) | 4  (7.5%) | 0.7837 |
|  | Vision | 106 (10.7%) | 27  (8.3%) | 26  (8.6%) | 11  (6.0%) | 4  (7.5%) | 0.2639 |
|  | Headache | 422 (42.5%) | 84  (25.8%) | 76  (25.2%) | 32 (17.5%) | 10  (18.9%) | **<0.001**  (0.2073) |
|  | Sensory | 116 (11.7%) | 37  (11.4%) | 33  (10.9%) | 18  (9.8%) | 6  (11.3%) | 0.9654 |
|  | GCS^2^ | 44  (4.4%) | 11  (3.4%) | 14  (4.6%) | 6  (3.3%) | 2  (3.8%) | 0.8751 |
|  | Incidental | 14  (1.4%) | 2  (0.6%) | 3  (1.0%) | 6  (3.3%) | 3  (5.7%) | **0.01483**  (0.0816) |
|  | Other | 139 (14.0%) | 39  (12.0%) | 43  (14.2%) | 23 (12.6%) | 9  17.0%) | 0.8037 |
| **Medication use** | | | | | | | |
| Steroid use | Recorded | 972 | 317 | 296 | 180 | 51 |  |
|  |  | 774 (79.6%) | 261 (82.3%) | 244 (82.4%) | 141 (78.3%) | 42  (82.4%) | 0.6441 |
| AED^3^ use | Recorded | 986 | 322 | 300 | 183 | 52 |  |
|  |  | 476 (48.3%) | 148 (46.0%) | 131 (43.7%) | 63 (34.4%) | 13  (25.0%) | **<0.001**  (0.1074) |
| **Tumour location on MRI** | | | | | | | |
| Tumour location | Recorded | 994 | 325 | 302 | 183 | 53 |  |
|  | Frontal | 374 (37.6%) | 136 (41.8%) | 117 (38.7%) | 50 (27.3%) | 14  (26.4%) | **0.0084**  (0.0856) |
|  | Parietal | 276  (27.8%) | 87  (27.7%) | 110  (36.4%) | 69  (37.7%) | 24  (45.3%) | **<0.001**  (0.1053) |
|  | Temporal | 439 (44.2%) | 136 (41.4%) | 127 (41.3%) | 80 (43.7%) | 23  (43.4%) | 0.9404 |
|  | Occipital | 78  (7.2%) | 33  (10.2%) | 28  (9.3%) | 15  (8.2%) | 8  (15.1%) | 0.3225 |
|  | Cerebellum | 16  (1.6%) | 3  (0.9%) | 2  (0.7%) | 3  (1.6%) | 1  (1.9%) | 0.6887 |
|  | Brainstem | 32  (3.2%) | 7  (2.2%) | 4  (1.3%) | 3  (1.6%) | 0  (0.0%) | 0.2032 |
|  | Multifocal | 239 (24.1%) | 80  (24.7%) | 86  (28.5%) | 43  (23.5%) | 12  (22.6%) | 0.5919 |
| **Molecular diagnostics** | | | | | | | |
| *MGMT*^4^ methylation | *MGMT*^4^ methylation reported | 994 | 325 | 302 | 183 | 53 |  |
|  | *MGMT*  Methylation ≥10% | 244 (24.5%) | 93  (28.6%) | 78  (25.8%) | 55  (30.1%) | 10  (18.9%) | 0.2661 |
| *EGFR*^5^ status | Reported | 956 | 318 | 299 | 181 | 52 |  |
|  | *EGFR*^5^ amplified | 166 (17.4%) | 44 (13.8%) | 33 (11.0%) | 24 (13.3%) | 7 (13.5%) | **<0.001**  (0.08937) |
|  | *EGFR* not amplified | 273 (28.6%) | 79 (24.8%) | 58 (19.4%) | 44 (24.3%) | 8 (15.4%) |  |
|  | *EGFR* not tested | 517 (54.1%) | 195 (61.3%) | 208 (69.6%) | 113 (62.4%) | 37 (71.2%) |  |
| Chr 7+/Chr 10- | Reported | 947 | 317 | 303 | 178 | 51 |  |
|  | Chr 7+/Chr 10- present | 75 (7.9%) | 14 (4.4%) | 14 (4.7%) | 5 (2.8%) | 0 (0.0%) | **<0.001**  (0.07961) |
|  | Chr 7+/Chr 10-  absent | 118 (12.5%) | 29 (9.1%) | 24 (8.0%) | 19 (9.9%) | 5 (9.4%) |  |
|  | Chr 7+/Chr 10-  not tested | 754 (79.6%) | 274 (86.4%) | 265 (88.9%) | 154 (86.5%) | 46 (92.0%) |  |
| *TERT*^6^*-*mutation | Reported | 972 | 322 | 301 | 180 | 52 |  |
|  | *TERT*^6^*-*mutation present | 370 (38.1%) | 94 (29.6%) | 72 (24.1%) | 49 (27.5%) | 11 (22.0%) | **<0.001**  (0.1173) |
|  | *TERT-*mutation  not present | 95 (9.8%) | 20 (6.3%) | 16 (5.3%) | 9 (5.0%) | 5 (9.4%) |  |
|  | *TERT-*mutation  not tested | 507 (52.2%) | 208 (65.2%) | 213 (70.6%) | 122 (67.4%) | 36 (67.9%) |  |
| **Surgical and oncological treatment** | | | | | | | |
| Surgical treatment | Surgery reported | 994 | 325 | 302 | 183 | 53 |  |
|  | Biopsy | 269  (27.1%) | 92  28.3%) | 116  (38.4%) | 86  (47.0%) | 26  (49.1%) | **<0.001**  (0.1557) |
|  | Resection | 725  (72.9%) | 233  (71.7%) | 186  (61.6%) | 97  (53.0%) | 27  (50.9%) |  |
| Extent of resection | Biopsy | 269  (27.1%) | 92  28.3%) | 116  (38.4%) | 86  (47.0%) | 26  (49.1%) | **<0.001**  (0.1225) |
|  | Resection <95% | 324  (32.6%) | 129  (39.7%) | 95  (31.5%) | 52  (28.4%) | 11  (20.8%) |  |
|  | Resection ≥95% | 401  (40.3%) | 104  (32.0%) | 91  (30.1%) | 45  (24.6%) | 16  (30.2%) |  |
| Oncological treatment by intensity | Surgery alone | 158 (15.9%) | 70  (21.5%) | 87  (27.5%) | 61  (33.9%) | 24  (45.3%) | **<0.001**  (0.223) |
|  | Intermediate | 168  (16.9%) | 74  (22.8%) | 88  29.1%) | 73  (40.6%) | 25  (47.2%) |  |
|  | Aggressive | 668  (67.2%) | 181  (55.7%) | 131  (43.4%) | 50  (27.3%) | 4  (7.5%) |  |
| Firstline oncological treatment by regimen | Treatment reported | 749 | 229 | 211 | 116 | 21 |  |
|  | Temozolomide | 24  (2.6%) | 9  (3.0%) | 6  (2.2%) | 4  (2.2%) | 7  (15.6%) | **<0.001**  (0.3189) |
|  | Hypofractionated RT | 57  (6.3%) | 39  (13.2%) | 34  (12.4%) | 47 (26.1%) | 10  (22.2%) |  |
|  | Hypofractionated CRT  (Perry *et al.*) | 58  (6.4%) | 40  (13.4%) | 94  (34.3%) | 42 (25.1%) | 3  (6.7%) |  |
|  | Conventionally fractionated CRT  (Stupp *et al.*) | 610 (67.4%) | 141 (47.1%) | 77  (27.5%) | 23 (12.8%) | 1  (2.2%) |  |
| **Treatment completion** | | | | | | | |
| Concurrent treatment | Completion reported | 682 | 189 | 136 | 55 | 5 |  |
|  | Not completed | 87  (12.8%) | 27  (14.3%) | 16  (11.8%) | 7  (12.7%) | 0  (0%) | 0.8796 |
|  | Completed | 595 (87.2%) | 162 (85.7%) | 120 (88.2%) | 48  (87.3%) | 5  (100%) |  |
| Adjuvant treatment | Completion reported | 575 | 156 | 104 | 46 | 11 |  |
|  | Not completed | 329 (57.3%) | 86 (55.2%) | 62 (58.6%) | 35 (76.0%) | 7  (63.6%) | 0.1281 |
|  | Completed | 246 (42.7%) | 70 (44.8%) | 42 (40.4%) | 11 (24.0%) | 4  (36.4%) |  |
| Adjuvant discontinuation reason | Reason reported | 329 | 86 | 62 | 35 | 7 |  |
|  | Toxicity  (haematological) | 20  (6.1%) | 5  (6.0%) | 6  (9.7%) | 1  (2.9%) | 0  (0%) | 0.1706 |
|  | Toxicity (non-haematological) | 58 (17.6%) | 23  (26.7%) | 17  (27.4%) | 11 (31.4%) | 2  (28.6%) |  |
|  | Progression | 251 (76.3%) | 58  (67.4%) | 39  (62.9%) | 23 (65.7%) | 5  (71.4%) |  |
| Stopped due to toxicity | Reason reported | 994 | 325 | 302 | 183 | 53 |  |
|  | No | 685 (68.9%) | 244 (75.1%) | 246 (81.5%) | 149 (81.4%) | 46 (86.8%) | **<0.001**  (0.1309) |
|  | Yes | 309  (31.1%) | 81 (24.9%) | 56  (18.5%) | 34  (18.6%) | 7  (13.2%) |  |
| **Additional treatments** | | | | | | | |
| 2nd Line Treatment | No | 373 (47.9%) | 145 (61.7%) | 147 (70.3%) | 88 (77.9%) | 26 (83.9%) | **<0.001**  (0.231) |
|  | Yes | 406 (52.1%) | 90 (38.3%) | 62 (29.7%) | 25 (22.1%) | 5  (16.1%) |  |
| 2nd Line Treatment Type | Surgery | 85 (21.1% | 22 (24.7%) | 8  (12.9%) | 4  (16.0%) | 2  (40.0%) | 0.7442 |
|  | Radiotherapy (no PTV overlap) | 7 (1.7%) | 4 (4.5%) | 1 (1.6%) | 1 (4.0%) | 0 |  |
|  | Radiotherapy (marginal PTV overlap) | 10 (2.5%) | 1 (1.1%) | 3 (4.8%) | 1 (4.0%) | 0 |  |
|  | Radiotherapy (reirradiation/full PTV overlap) | 13 (3.2%) | 3 (3.4%) | 1 (1.6%) | 1 (4.0%) | 0 |  |
|  | Systemic therapy | 279 (69.2%) | 55 (61.8%) | 49 (79.0%) | 18 (72.0%) | 3 (60.0%) |  |
|  | Clinical trial | 9 (2.2%) | 4 (4.5%) | 0 | 0 | 0 |  |
| 3rd Line Treatment | No | 158 (56.0%) | 40 (71.4%) | 22 (55.0%) | 14 (87.5%) | 1 (33.3%) | **0.026**  (0.167) |
|  | Yes | 124 (44.0%) | 16 (28.6%) | 18 (45.0%) | 2 (12.5%) | 2 (66.7%) |  |
| 3rd Line Treatment Type | Surgery | 12 (9.7%) | 1 (6.3%) | 1 (5.6%) | 0 | 0 | **0.044**  (0.222) |
|  | Radiotherapy (no PTV overlap) | 5 (4.0%) | 0 | 2 (11.1%) | 0 | 0 |  |
|  | Radiotherapy (marginal PTV overlap) | 2 (1.6%) | 0 | 0 | 0 | 1 (50.0%) |  |
|  | Radiotherapy (reirradiation/full PTV overlap) | 9 (7.3%) | 1 (6.3%) | 2 (11.1%) | 0 | 0 |  |
|  | Systemic therapy | 92 (74.2%) | 14 (87.5%) | 13 (72.2%) | 2 (100%) | 1 (50.0%) |  |
|  | Clinical trial | 4 (3.2%) | 0 | 0 | 0 | 0 |  |
| 4th Line Treatment | No | 43 (58.1%) | 4 (50.0%) | 9 (75.0%) | 0 | 1 (100%) | 0.275 |
|  | Yes | 31 (41.90) | 4 (50.0%) | 3 (25.0%) | 2 (100%) | 0 |  |
| 4th Line Treatment Type | Surgery | 3 (9.7%) | 0 | 0 | 0 | 0 | NA |
|  | Radiotherapy (no PTV overlap) | 0 | 0 | 0 | 0 | 0 |  |
|  | Radiotherapy (marginal PTV overlap) | 1 (3.2%) | 0 | 0 | 0 | 0 |  |
|  | Radiotherapy (reirradiation/full PTV overlap) | 3 (9.7%) | 1 (25.0%) | 0 | 0 | 0 |  |
|  | Systemic therapy | 24 (77.4%) | 3 (75.0%) | 3 (100%) | 2 (100%) | 0 |  |
|  | Clinical trial | 0 | 0 | 0 | 0 | 0 |  |

Percentages represent proportion of patients with reported variable. ^1^Performance status documented at diagnosis. ^2^Presenting complaint dichotomized; ^3^GCS Glasgow Coma Score; ^4^AED Antiepileptic Drug; ^5^*MGMT* O^6^-methylguanine-DNA methyltransferase status dichotomized as <10%, ≥10%; ^6^*EGFR* Epidermal Growth Factor Receptor gene; ^7^*TERT* Telomerase Reverse Transcriptase gene; ^8^Chemoradiotherapy.

**Supplemental Figure 1:** Concurrent therapy treatment termination due to toxicity and mean PTV compared between age categories <65 and ≥65 years.
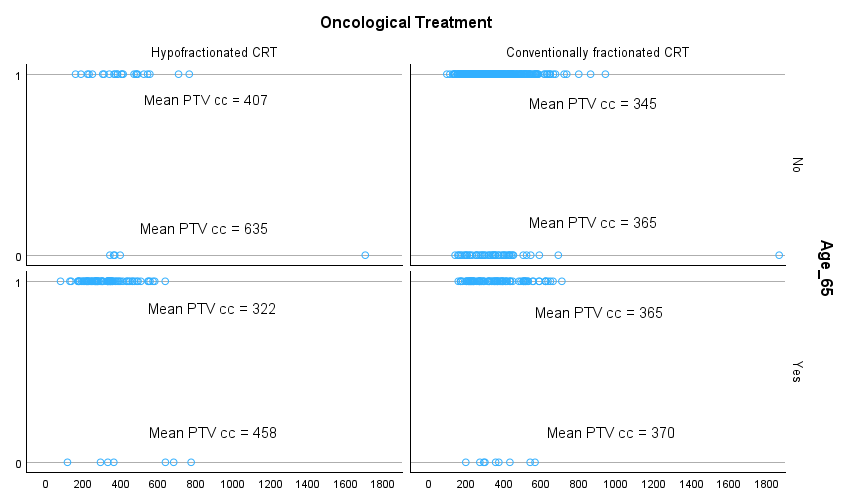


| Oncological Treatment | Completed concurrent chemotherapy | Age <65 | | | Age >65 | | |
| --- | --- | --- | --- | --- | --- | --- | --- |
|  |  | Planning Target Volume (cc) | | | | | |
|  |  | Mean | Median | Range | Mean | Median | Range |
| Hypofractionated Chemoradiotherapy | No | 635 | 369 | 343 - 1704 | 458 | 364 | 117 - 776 |
|  | Yes | 407 | 395 | 160 - 766 | 322 | 303 | 80 - 638 |
| Conventionally fractionated chemoradiotherapy | No | 365 | 339 | 140 - 1867 | 370 | 356 | 197 - 566 |
|  | Yes | 345 | 324 | 96 - 941 | 365 | 351 | 157 - 709 |

**Supplemental Figure 2:** Adjuvant therapy treatment termination due to toxicity and mean PTV compared between age categories <65 and ≥65 years.


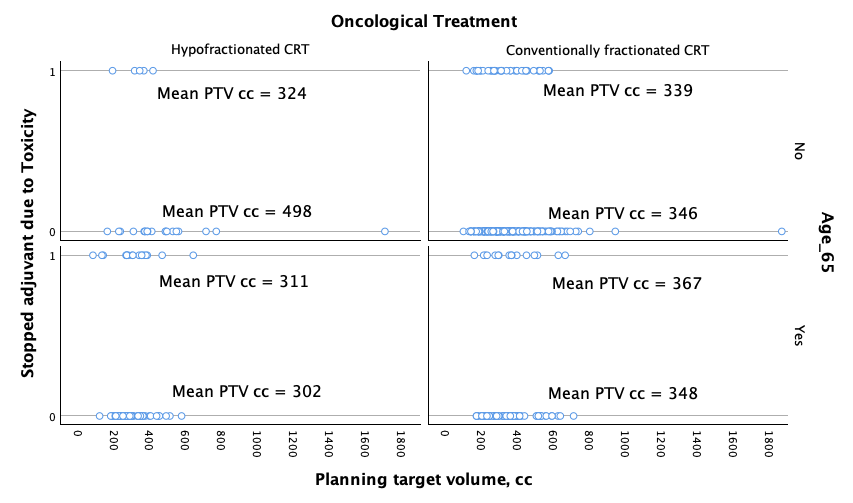


| Oncological Treatment | Stopped due to toxicity | Age <65 | | | Age >65 | | |
| --- | --- | --- | --- | --- | --- | --- | --- |
|  |  | Planning Target Volume (cc) | | | | | |
|  |  | Mean | Median | Range | Mean | Median | Range |
| Hypofractionated Chemoradiotherapy | No | 498 | 407 | 160 - 1704 | 302 | 285 | 117 - 573 |
|  | Yes | 324 | 340 | 189 - 414 | 311 | 302 | 80 - 638 |
| Conventionally fractionated chemoradiotherapy | No | 346 | 319 | 96 - 1867 | 348 | 324 | 169 - 709 |
|  | Yes | 339 | 309 | 112 - 573 | 367 | 357 | 157 - 662 |

**Supplemental Figure 3:** Stacked bar charts summarizing second-, third- and fourth-line systemic therapies, received by patients following recurrence following planned concurrent/adjuvant therapy, stratified by age group.


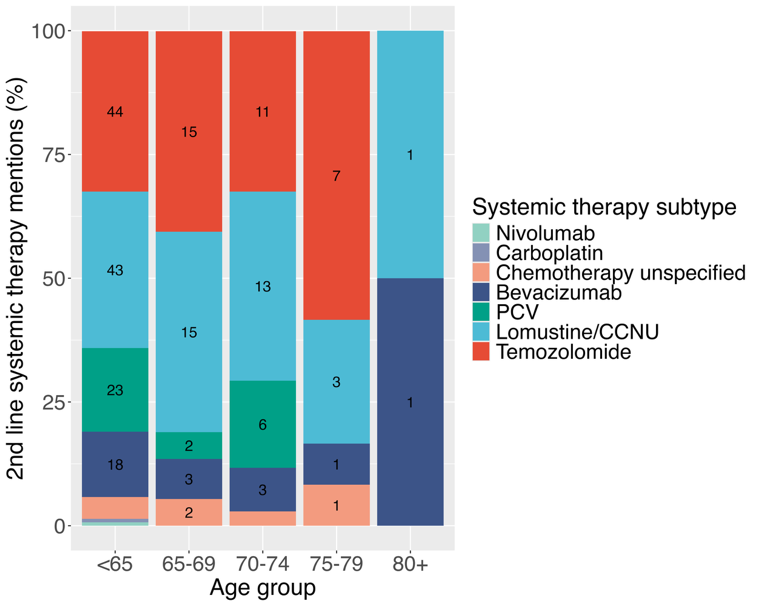

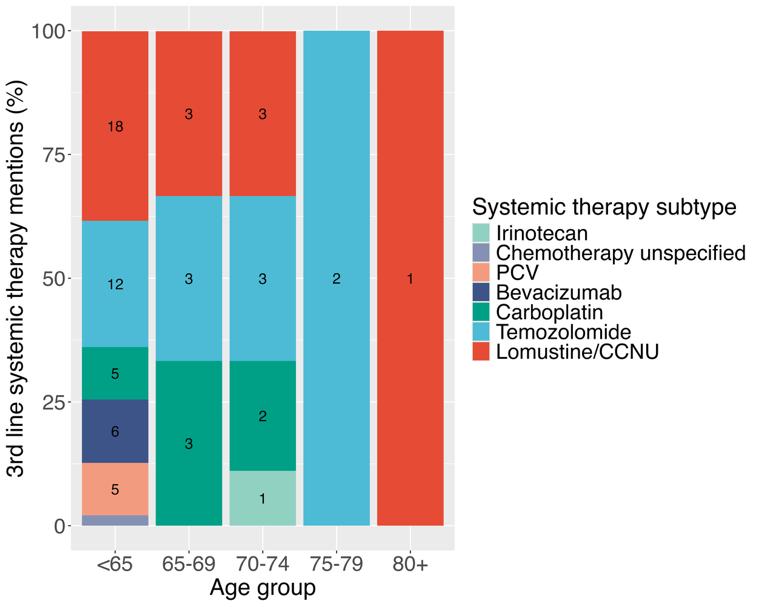

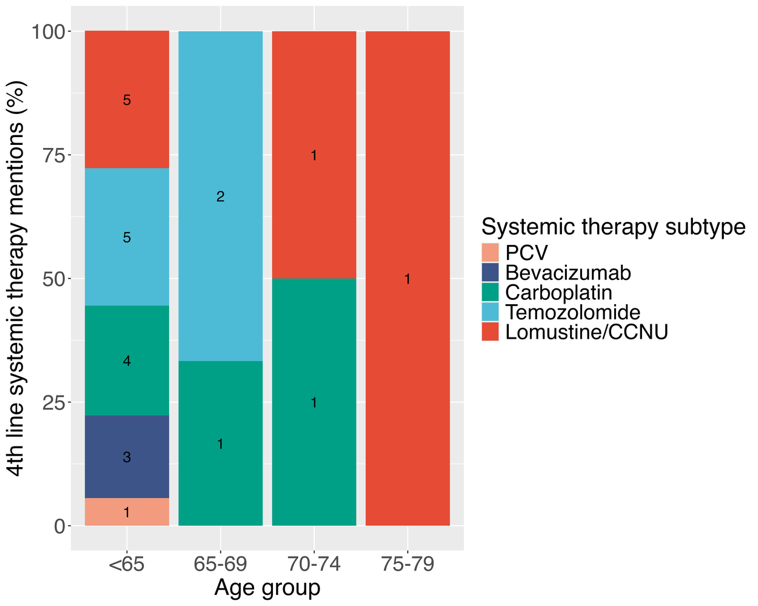


**Supplemental Table 4:** Survival data stratified by treatment regimen intensity and age category.

| Treatment Group | Age Group | Median overall survival (months) | Lower – Upper bound 95% CI (months) | | Chi-square (Degrees of freedom) | Significance p value |
| --- | --- | --- | --- | --- | --- | --- |
| Surgery only (Biopsy) | <65 | 1.74 | 1.40 | 2.08 | 3.381 (4) | 0.496 |
|  | 65-69 | 1.71 | 1.16 | 2.26 |  |  |
|  | 70-74 | 2.07 | 1.57 | 2.57 |  |  |
|  | 75-79 | 2.46 | 1.25 | 3.68 |  |  |
|  | ≥80 | 3.22 | 1.28 | 5.15 |  |  |
|  | Overall | 2.00 | 1.77 | 2.24 |  | |
| Surgery only (Resection) | <65 | 2.69 | 1.15 | 4.24 | 3.313 (4) | 0.507 |
|  | 65-69 | 2.43 | 1.00 | 3.86 |  |  |
|  | 70-74 | 3.06 | 1.95 | 4.16 |  |  |
|  | 75-79 | 3.29 | 2.18 | 4.39 |  |  |
|  | ≥80 | 5.12 | 2.85 | 7.40 |  |  |
|  | Overall | 3.15 | 2.45 | 3.86 |  | |
| Intermediate (Biopsy) | <65 | 7.79 | 6.45 | 9.12 | 3.819 (4) | 0.431 |
|  | 65-69 | 6.34 | 3.32 | 9.36 |  |  |
|  | 70-74 | 5.26 | 3.14 | 7.37 |  |  |
|  | 75-79 | 4.93 | 3.02 | 6.83 |  |  |
|  | ≥80 | 5.32 | 2.42 | 8.22 |  |  |
|  | Overall | 6.64 | 5.43 | 7.84 |  | |
| Intermediate (Resection) | <65 | 12.71 | 11.88 | 13.54 | 25.534 (4) | <0.001 |
|  | 65-69 | 8.48 | 7.65 | 9.30 |  |  |
|  | 70-74 | 8.05 | 5.53 | 10.57 |  |  |
|  | 75-79 | 8.41 | 7.49 | 9.33 |  |  |
|  | ≥80 | 10.05 | 7.10 | 13.01 |  |  |
|  | Overall | 9.56 | 8.63 | 10.49 |  | |
| Aggressive (Biopsy) | <65 | 12.12 | 10.29 | 13.96 | 6.001 (3) | 0.112 |
|  | 65-69 | 8.51 | 7.48 | 9.54 |  |  |
|  | 70-74 | 9.79 | 7.92 | 11.66 |  |  |
|  | 75-79 | 8.38 | 1.74 | 15.01 |  |  |
|  | ≥80 | - | - | - |  | |
|  | Overall | 11.07 | 9.85 | 12.29 | 6.698 (4) | 0.153 |
| Aggressive (Resection) | <65 | 16.79 | 15.70 | 17.88 |  |  |
|  | 65-69 | 17.08 | 14.36 | 19.81 |  |  |
|  | 70-74 | 15.80 | 13.18 | 18.43 |  |  |
|  | 75-79 | 14.65 | 9.85 | 19.46 |  |  |
|  | ≥80 | 13.11 | 0.00 | 30.25 |  |  |
|  | Overall | 16.62 | 15.68 | 17.54 |  | |

**Supplemental Table 5:** Survival data stratified by treatment for patients receiving hypofractionated chemoradiotherapy or conventionally fractionated chemoradiotherapy for different age categories (<65, 65-69 and ≥70 years of age) and all performance status (0-4).

| Case Processing Summary | | | | | |
| --- | --- | --- | --- | --- | --- |
| Oncological Treatment | Age group | Total N | N of Events | Censored | |
|  |  |  |  | N | Percent |
| Hypofractionated CRT | <65 | 58 | 52 | 6 | 10.3% |
|  | 65-69 | 40 | 35 | 5 | 12.5% |
|  | 70+ | 139 | 125 | 14 | 10.1% |
|  | Overall | 237 | 212 | 25 | 10.5% |
| Conventionally fractionated CRT | <65 | 610 | 491 | 119 | 19.5% |
|  | 65-69 | 141 | 106 | 35 | 24.8% |
|  | 70+ | 46 | 38 | 8 | 17.4% |
|  | Overall | 797 | 635 | 162 | 20.3% |
| Overall | Overall | 1034 | 847 | 187 | 18.1% |

| Medians for Survival Time in Months | | | | | |
| --- | --- | --- | --- | --- | --- |
| Oncological Treatment | Age group | Median |  | | |
|  |  | Estimate | Std. Error | 95% Confidence Interval | |
|  |  |  |  | Lower Bound | Upper  Bound |
| Hypofractionated CRT | <65 | 9.2 | 0.9 | 7.5 | 10.9 |
|  | 65-69 | 9.6 | 1.2 | 7.2 | 12 |
|  | 70+ | 13.3 | 0.8 | 11.7 | 14.8 |
|  | Overall | 11.8 | 0.6 | 10.6 | 12.9 |
| Conventionally fractionated CRT | <65 | 16.8 | 0.5 | 15.7 | 17.8 |
|  | 65-69 | 16.9 | 1.5 | 13.9 | 19.8 |
|  | 70+ | 13 | 2.2 | 8.6 | 17.4 |
|  | Overall | 16.6 | 0.5 | 15.6 | 17.6 |
| Overall | Overall | 15.3 | 0.5 | 14.3 | 16.2 |

| Overall Comparisons | | | | |
| --- | --- | --- | --- | --- |
| Oncological Treatment | Chi-Square | df | Sig. | |
| Hypofractionated CRT | Log Rank (Mantel-Cox) | 4.679 | 2 | .096 |
| Conventionally fractionated CRT | Log Rank (Mantel-Cox) | 5.931 | 2 | .052 |

**Supplemental Table 6:** Survival data stratified by treatment for patients receiving hypofractionated chemoradiotherapy or conventionally fractionated chemoradiotherapy for different age groups (<65, 65-69 and ≥70 years of age) and performance status groups 0-1.

| Case Processing Summary | | | | | |
| --- | --- | --- | --- | --- | --- |
| Oncological Treatment | Age group | Total N | N of Events | Censored | |
|  |  |  |  | N | Percent |
| Hypofractionated CRT | <65 | 41 | 37 | 4 | 9.8% |
|  | 65-69 | 25 | 21 | 4 | 16.0% |
|  | 70+ | 106 | 96 | 10 | 9.4% |
|  | Overall | 172 | 154 | 18 | 10.5% |
| Conventionally fractionated CRT | <65 | 497 | 403 | 94 | 18.9% |
|  | 65-69 | 108 | 84 | 24 | 22.2% |
|  | 70+ | 37 | 34 | 3 | 8.1% |
|  | Overall | 642 | 521 | 121 | 18.8% |
| Overall | Overall | 814 | 675 | 139 | 17.1% |

| Medians for Survival Time | | | | | |
| --- | --- | --- | --- | --- | --- |
| Oncological Treatment | Age group | Median |  |  |  |
|  |  | Estimate | Std. Error | 95% Confidence Interval | Estimate |
|  |  |  |  | Lower Bound | Upper Bound |
| Hypofractionated CRT | <65 | 9.8 | 1.1 | 7.8 | 11.9 |
|  | 65-69 | 11.0 | 2.0 | 7.0 | 15.0 |
|  | 70+ | 13.9 | 0.9 | 12.1 | 15.7 |
|  | Overall | 12.1 | 0.8 | 10.4 | 13.7 |
| Conventionally fractionated CRT | <65 | 16.9 | 0.6 | 15.7 | 18.0 |
|  | 65-69 | 17.1 | 1.6 | 13.9 | 20.3 |
|  | 70+ | 13.0 | 2.1 | 8.9 | 17.1 |
|  | Overall | 16.8 | 0.5 | 15.8 | 17.8 |
| Overall | Overall | 15.9 | 0.5 | 14.8 | 17.0 |

| **Overall Comparisons** | | | | |
| --- | --- | --- | --- | --- |
| Oncological Treatment | Chi-Square | df | Sig. | |
| Hypofractionated CRT | Log Rank (Mantel-Cox) | 4.135 | 2 | .127 |
| Conventionally fractionated CRT | Log Rank (Mantel-Cox) | 7.043 | 2 | .030 |
